# Supplementary material for: Are carbon emissions trading and green financial instruments synergistic? -Comprehensive quantitative research based on content analysis
Source: PLoS One. 2024 Mar 7;19(3):e0298601. doi: 10.1371/journal.pone.0298601 (PMC10919601; doi:10.1371/journal.pone.0298601)
Supplement: S1 Data — (ZIP) [file pone.0298601.s001.zip › Data/地级市Z_F工作报告.zip!/║╙─╧/╓ú╓▌╩╨/└┤╫╘╣1⁄2╓┌║┼í╛┬φ┐╦╩2╛▌═°í┐_2018.docx]

各位代表：

现在，我代表市人民政府向大会报告工作，请予审议，并请各位政协委员和列席人员提出意见。

一、本届政府工作回顾

本届政府任期以来，面对复杂的国际国内形势和繁重的改革发展任务，在市委的坚强领导下，全市上下坚持以习近平新时代中国特色社会主义思想为指导，以党的十八大、十九大精神和习近平总书记调研指导河南和郑州工作时的重要讲话为统领，全面落实党中央、国务院和省委、省政府的各项决策部署，深度融入“一带一路”建设，大力实施“三区一群”战略，坚持“四重点一稳定一保证”工作总格局，稳增长、促改革、调结构、惠民生、防风险，经济社会持续平稳健康发展，较好完成了市第十四届人民代表大会确定的主要目标任务，解决了许多长期想解决而没有解决的难题，办成了许多过去想办而没有办成的大事，一大批事关全局的节点性、标志性、奠基性大事要事取得重大突破，郑州在实现中原崛起、支撑中部崛起、服务全国大局中的地位和作用显著增强，国家政策叠加优势在全国城市中屈指可数，城市影响力、辐射力、带动力和美誉度持续提升。郑州发展迈入新时代，踏上新征程！

——国家中心城市建设迈出坚实步伐。抢抓国家支持郑州建设国家中心城市重大机遇，制定实施《郑州建设国家中心城市行动纲要（2017—2035年）》，进一步明确了郑州在全国乃至全球城市发展格局中的新定位，作出了“三个阶段”的战略安排，描绘了到本世纪中叶“建成具有全球影响力城市”的发展愿景，开启了郑州向全国乃至全球城市体系更高层级城市迈进的新征程！

——综合实力大幅攀升。“十二五”规划圆满完成，“十三五”规划顺利实施，全市地区生产总值连跨7000亿元、8000亿元、9000亿元三个台阶，2017年达到9130亿元，经济总量在全国27个省会城市中由第8位提升至第7位，全省经济首位度由19.3%提高到20.3%；今年上半年，生产总值达到4611.4亿元、增长8.5%，增速分别比全国、全省高出1.7个和0.7个百分点。预计今年地区生产总值将突破1万亿元，5年年均增长约9%、人均地区生产总值将突破10万元大关。2017年地方财政一般公共预算收入跨越千亿元大关、达到1056.7亿元，是2013年的1.6倍、年均增长13%，占全省比重的31.1%；上半年地方财政一般公共预算收入627亿元、增长15.9%，高于全省1.4个百分点；预计今年将达到1150亿元。

——开放创新取得重大突破。国家首个航空港经济综合实验区“五年成规模”目标全面实现。中国（郑州）跨境电子商务综试区、河南自贸区、郑州国家通用航空产业综合示范区先后获得国家批复并启动建设。各类口岸和海关特殊监管区建设取得重大突破，成为全国拥有功能性口岸最多的内陆城市。郑洛新国家自主创新示范区成为国家战略，创新创业综合体建设、“智汇郑州人才工程”加快推进，国家专利审查协作河南中心等一批国家级功能平台和科研机构成功落地，科技进步贡献率达到62%。

——现代化国际性立体综合交通枢纽地位持续强化。郑州被明确为全国十二个最高等级的国际性综合交通枢纽之一，机场二期建成投用、三期启动建设，航线由2013年的143条增加到228条，客货运量分别由2013年的1314万人次、25.6万吨增长到2017年的2430万人次、50.3万吨，年均分别增长16.6%、18.4%，今年上半年增速分别跃居全国大型机场第1和第2位。“米”字形高铁加快成形，郑万、郑济、郑合高铁、郑州南站及配套工程建设进展顺利，郑徐高铁和郑开、郑焦、郑机城铁开通运营。

——转型发展气势强劲。三次产业比由2013年的2.3∶52.1∶45.6调整为2017年的1.7∶46.5∶51.8，实现了三产比重超过一产和二产合计、战略性新兴产业比重超过高耗能行业两大历史性突破。成功获批“中国制造2025”试点示范城市，入选国家信息消费示范城市、互联网骨干直连点城市，建成互联网国际通信专用通道。规模以上工业单位增加值能耗年均下降13.5%，超额完成目标任务。高新技术产值年均增长12.5%。

——生态文明建设成效明显。全市新增绿地5730万平方米，森林覆盖率达到33.6%，贾鲁河综合治理工程全面推进，城区河道生态供水由年均1亿立方米增加到3亿立方米，河流水量水质明显改善；PM10、PM2.5浓度比2013年分别下降30.6%和38.9%，空气优良天数明显增多，超额完成“大气十条”五年考核目标。成功举办第十一届中国（郑州）国际园林博览会，入选国家生态文明先行示范区、综合管廊试点城市，顺利通过国家节水型城市建设复查和全国水生态文明城市建设试点验收。

——群众生活质量全面提升。脱贫攻坚数据质量指数达到99.99%，全市省级贫困人口由97439人减少至5731人，290个贫困村全部脱贫退出。新增城镇就业67.8万人、农村劳动力转移就业42.4万人。新增中小学学位20.7万个、幼儿园学位5.7万个。预计今年城镇居民人均可支配收入将达到39110元、农村居民人均可支配收入达到21670元，5年年均增长分别为8%、9.1%。

我们重点抓了以下工作：

（一）抓机遇重谋划，国家中心城市建设开篇布局。国家《促进中部地区崛起“十三五”规划》明确提出，支持郑州建设国家中心城市，提升了格局，改善了预期，提振了信心。坚持把党的十九大精神和中央各项决策精神、省委工作部署与郑州实际相结合，认真贯彻市委十一届四次、五次、六次、七次全会精神，以国家中心城市高质量建设和经济社会高质量发展为主线，紧紧围绕“六大功能定位、十大主攻方向、九大支撑性工程、六大基础性工作”，全面落实建设国家中心城市的目标定位、发展路径、基本原则、工作思路、主要任务；建立并实施了总投资4.37万亿元的国家中心城市建设重大项目库，成立了高规格领导机构，理顺了工作推进机制，明确了43项重点任务；《郑州市城市总体规划（2018—2035年）》编制工作加快推进，土地利用总体规划、“1+4”郑州大都市区空间规划及4个专项规划编制完成；国家中心城市高质量建设规划和实践体系初步构建，各项工作的推进蹄疾步稳。

（二）强措施稳运行，经济保持持续健康发展。面对经济下行压力不断加大的严峻形势，我们把稳增长作为抓好工作的首要任务，把解决阶段突出矛盾和夯实长远发展基础相结合，积极化解经济运行中出现的矛盾和问题。供给侧结构性改革深入推进。“三去一降一补”成效显著，重点行业过剩产能有效化解，退出钢铁产能165万吨、压减煤炭产能1573万吨。坚持“房子是用来住的，不是用来炒的”定位，不断完善房地产调控措施，房地产业平稳健康发展，市区商品住房去库存任务圆满完成。积极稳妥去杠杆，控制债务规模、增加股权融资，政府、金融机构杠杆率有效降低，风险得到有效管控。营改增全面落实，企业降本减负有力有效，累计为企业减负约1100亿元。投资结构不断优化。全市固定资产投资由2013年的4400亿元增加到2017年的7573.4亿元，年均增长14.6%。民间投资由2013年的3165.4亿元增加到2017年的4951.9亿元，占固定资产投资比重65.3%，年均增长11.8%。要素保障不断强化。积极探索科学用地方式和弹性供地机制，重大项目用地得到优先保障。充分发挥政府资金杠杆作用，设立郑州国家中心城市产业发展千亿元基金；累计争取国家、省资金56.9亿元，国家专项建设基金55.18亿元；争取国际金融组织和外国政府贷款3.44亿美元，境外发债33.91亿美元，全市挂牌上市公司总数从37家增加到204家，占全省40%以上。

（三）调结构促转型，现代产业体系加快构建。实施转型攻坚发展“1+5+1”政策体系，围绕12个重点转型产业出台推进高质量发展专案，产业结构持续优化。工业综合实力显著增强。2017年规模以上工业总产值超过1.6万亿元，继续保持中部省会城市首位、跨入全国第一方阵。大力发展战略新兴产业，改造提升传统优势产业，推动制造业智能化、服务化，汽车及装备制造等四大战略性产业占比47.1%，比2013年提高3.2个百分点；煤炭等六大高耗能行业占比40.5%，比2013年下降4.3个百分点；国家新型工业化产业示范基地达到3个。今年上半年七大主导产业增加值增速高于全市工业2.2个百分点、贡献率达94.1%。积极承接产业转移，成功引进上海汽车、中铁装备、中车轨道、比克电池等一批重大产业项目。累计完成工业投资超过6000亿元，竣工项目近600个。千亿级主导产业集群达到6个，手机总出货量超过全球七分之一，整车产能达到120万辆。建筑业转型发展取得新突破，增加值占GDP比重从5.7%提升至7.1%，今年上半年产值同比增加21.5%。现代服务业提质增效。国家区域性金融中心地位日益凸显，郑州在全国28个区域金融中心城市中的排名跃升至第10位，金融业增加值占GDP比重从7.9%上升至10.8％，存贷款余额均居全国省会城市第6位。12家全国性股份制商业银行全部集聚郑州，郑东新区金融集聚核心功能区迈上新台阶，龙湖金融岛加快规划建设，国家区域性现代金融中心载体再获突破，全球首个鲜果期货品种“苹果期货”在郑商所上市。内贸流通体制改革取得突破，电子商务成为发展新引擎，2017年全市电子商务交易额6002亿元、今年上半年增速达21.1%。物流业发展态势良好，今年上半年完成增加值313.8亿元、增长9.6%，国家级示范物流园区达到3个。国家区域性会展中心城市地位基本确立，会展业综合竞争力位居全国前列。文化创意旅游业快速发展，深圳华强等一批重大项目建成运营；以创建国家全域旅游示范区为带动，旅游接待总人数、总收入年均分别增长12.7%、12.8%。大力推动市场外迁和传统商贸业升级，中心城区及大围合区域累计完成外迁、提升市场221家，新建和提升改造新型标准化农贸市场50家。都市生态农业稳步发展。持续强化农业综合生产能力、加快农业结构调整，建成高标准农田32万亩、都市生态农业示范园15万亩，新发展环城都市生态农业20万亩、湿地农业1.1万亩；农产品质量安全监测合格率持续稳居全国前列。国家级、省级农业产业化龙头企业达到67家、市级以上农业产业化集群达到30个；美丽乡村和特色田园乡村项目建设步伐加快，全市休闲农业和乡村旅游星级企业达到61家；首届全国农业品牌大会、首届“中国农民丰收节”河南主会场活动成功举办，一二三产业融合发展水平位居全国大中城市前列。非公有制经济健康发展。2017年，我市非公有制经济总量达到6620亿元，占GDP比重72.5%、较2013年底提高2.8个百分点；完成税收581亿元，占全市税收总额的75%，较2013年底增加172亿元、占比提高2.6个百分点；非公有制企业11.5万家，从业人员217万人，较2013年底分别增加3.6万家、25万人，非公有制经济在经济社会发展中的作用更加凸显。

（四）提品质扩成效，城市功能和承载能力持续提升。坚持以人为核心，推动新型城镇化工作重心由“以拆开路、以拆促建”向“以建为主、提升品质、扩大成效”的重大转变，城乡建设全面提速，城乡一体化步伐不断加大，市县两级城市建成区面积达到830平方公里，全市城镇化率今年将达到73.5%，比2013年提高6.4个百分点。“畅通郑州”工程深入推进。轨道交通运营总里程达到93.6公里、累计客运量突破7亿人次；轨道交通2号线二期、3号线、5号线等加快建设，在建里程达到218公里。三环快速路、京广快速路、陇海快速路、农业快速化工程（不含铁路代建部分）等一批重大工程及407条支线路网建成通车，金水路西延、四环线及大河路快速化工程加快建设，中心城区“井字+环线”快速路网体系已经形成，道路“微循环”更加通畅。市域快速通道建成10条360公里，新建成环城高速出入口9个、我市环城高速出入口达到24个。豫A牌小客车免费通行绕城高速政策取得良好效果。“公交都市”创建进展顺利，快速公交成网率全国领先。全国主要城市拥堵指数排名从2014年第四季度的第9位降至2018年第二季度的第54位。百城建设提质工程深入推进。各县（市）规划成果编制和评审、控制性详细规划编制、专项规划编制有效推进。抓好以绿荫城、以水润城、以文化城、以业兴城“四篇文章”，加快实施老旧片区建设提质和示范区建设，已谋划项目1781个、总投资12652亿元，今年上半年实施项目1380个，完成投资722亿元。群众居住和生产生活环境持续改善。大棚户区改造加速推进，2014年以来累计动迁村庄732个，规划安置区（项目）353个，回迁群众74.9万人，郑州基本告别了中心城区城中村的时代。全面实施安置房、保障房网签工作，累计突破12万套。城市综合承载能力不断提升。成功获批全国第二批地下综合管廊建设试点城市、全国城市设计试点城市。城市综合执法体制改革持续深化，城市精细化管理三年行动计划全面启动；围绕“治脏、治乱、治差”，全面推行“路长制”，大力开展道路交通秩序、背街小巷和老旧社区综合整治，城市形象较大提升。新建公共停车泊位12.5万个。完成嵩山路等30条道路大修及76条道路（路段）中修工程。南水北调郑州段配套工程建成投运，受益人口680万人。刘湾水厂等相继建成投用，日供水能力比2013年底净增76万吨、达到177万吨。荥阳国电引热入郑等工程建成投用，集中供热普及率由不足50%提升至80%。南三环污水处理厂等相继投用，污水日处理能力比2013年底净增130万吨，达到190万吨，中心城区基本实现污水全收集全处理。220个规划保留村生活污水集中处理设施全面开建。四年多来解决了82.1万农村居民饮水不安全问题。生活垃圾分类、“厕所革命”、楼顶整治、游园微公园建设稳步推进。美丽乡村建设持续推进。新建改建农村道路1075公里；创建国家级生态乡镇7个、省级生态乡镇50个、省级生态村246个。

（五）抓改革促创新，发展新动能加快培育。深化改革多点发力。以“三个率先”为导向，聚焦落实标志性、关键性改革举措，发展内生动力进一步释放。“放管服”改革全面推开。实施“两集中、两到位”改革，内设审批处室由117个削减至37个，形成了一个窗口对外、一个机构履职、一枚印章签批的“三个一”审批模式。开展“五单一网”制度改革，建立权责清单，3414个审批事项在市、县、乡、村四级联动政务服务网系统办理。推行“一口受理”新机制，开展“减证便民”专项行动，累计取消繁文缛节和不必要证明事项364项，对56项保留证明实行清单管理，试点推行便民服务“同城通办”，缓解了群众“办事繁”“跑多趟”问题。启动“最多跑一次”改革，扎实做好省“三级十同”试点，审批服务事项和公共服务事项梳理工作稳步推进。大力推进不动产登记改革，有效解决各类遗留问题，受到住建部充分肯定。扎实推进“互联网+政务服务”，“i郑州”APP上线运行。商事登记制度改革稳步推进。全面启动“三十五证合一”，深入推动“双随机、一公开”监管全覆盖，累计新登记各类市场主体45.9万户，年均增长19.1%，河南自贸区郑州片区新注册企业3.3万家。全市市场主体突破100万家，新办企业审批服务时限压缩至4个工作日。国企改革持续深化。持续完善“1+N”政策体系，积极推进市管企业分类改革，基本完成市管企业公司制改制，市属企业剥离办社会职能工作有序进行。财税体制改革和投融资体制改革扎实开展。以贯彻落实新预算法为核心，完善政府预算体系，实行中期财政规划管理，持续规范政府债务管理，市本级投资三年滚动计划储备库初步建立，项目决策程序进一步规范。深化城乡规划土地建设管理“六统一”改革、投融资体制改革，政府性资源配置的市场化程度进一步提升。推广政府和社会资本合作（PPP）模式工作受到国务院表彰。农村改革不断深化。土地承包经营权确权登记颁证圆满完成，集体资产清产核资工作全面展开，集体经营性资产股份合作制改革稳步推进。同时，信用体系建设框架基本建立，信用环境显著改善。

创新驱动战略深入实施。自主创新示范区建设成效明显，高新区管理体制与人事薪酬制度改革基本完成，中原科创谷整体规划加快推进，国家质检中心郑州综合检测基地一期正式入驻，全国第四家商标审查中心——郑州商标审查协作中心正式落户，国家知识产权集聚发展示范区、国家技术转移郑州中心加快建设。以智慧岛为核心吸引华为、浪潮等一批龙头企业落地，国家大数据综合试验区建设成效明显。全省唯一基金注册地中原基金岛管理基金规模达1800亿元、入驻企业79家。以战略合作方式成立中国社科院郑州研究院，引进中科院过程所郑州分所、郑州轨道交通信息技术研究院等一批科研院所。发布“智汇郑州”人才政策30条，引进高层次创新创业人才524人。20个创新创业综合体投用585万平方米、累计入驻企业3737家。郑开双创走廊重大工程规划建设有序推进。我市成功获批国家促进科技和金融结合试点。

（六）扩开放植优势，内陆开放高地加速形成。大力实施开放带动战略，深度融入“一带一路”，加快建设“空中丝绸之路”“陆上丝绸之路”“网上丝绸之路”，无缝衔接“海上丝绸之路”，连通境内外、辐射东中西的物流通道枢纽地位进一步凸显，“买全球、卖全球”格局初步形成。2017年全市进出口总值完成596.4亿美元，是2013年的1.4倍，占全省的76.7%。招商引资规模持续扩大，四年来全市累计引进资金达到7914.4亿元；“走出去”步伐不断加快，全市对外投资中方协议出资额累计达到44.5亿美元。航空港实验区建设取得重大进展。智能终端产业入驻企业208家、投产66家，2017年手机产量接近3亿部，全球重要的智能终端基地初步形成。郑州新郑综合保税区全部建成，进出口总额稳居全国第一位，第二综保区成功获批。“引智试验区”“人民币创新试点”等国家战略平台相继落地。区域双创示范基地建设成效明显，受到国务院表彰。河南自贸区郑州片区建设加快推进。五大改革专项服务体系初步构建，省级确定的160项改革创新试点任务和市定的96项改革事项完成总任务的82%，形成原产地证书“信用签证”、政银合作直通车、“多式联运一单制”和跨境电商正面监管模式等多项创新举措。EWTO核心功能集聚区启动建设；2017年跨境电商进出口总额达到69.1亿美元、今年上半年增长36.7%；郑州成为全球跨境电商大会永久会址城市。汽车整车、肉类、水果、冰鲜水产品、食用水生动物指定口岸业务量稳步提升；郑州铁路口岸开展汽车平行进口试点，郑州进境粮食指定口岸经开和新郑两个查验区获批运营，邮政口岸成为全国国际邮件重要集疏口岸，郑州成为全国第四个中欧班列运邮试点城市。中欧班列实现每周“去八回八”常态化高频次运营，累计开行超过1300班；今年上半年开行300班，班列货运总量和满载率、境内外集疏分拨范围均居中欧班列前列。国内外影响力大幅提升。上合组织政府首脑（总理）理事会第十四次会议、国际旅游城市市长论坛、黄帝故里拜祖大典、郑州航展等重大活动成功举办，获批为中欧区域政策合作案例地区并成功承办第13次中欧区域政策合作研讨会，卢森堡旅游签证（郑州）便捷服务平台揭牌运营。

（七）重防治促统筹，生态文明建设扎实推进。牢固树立“绿水青山就是金山银山”的理念，坚持“大生态、大环保、大格局、大统筹”，以森林、湿地、流域、农田、城市五大生态体系为着力点，坚决打好“蓝天”“碧水”“净土”保卫战。“蓝天工程”深入实施。在全国地级市率先发布《“蓝天”工程白皮书》，获批国家北方地区冬季清洁取暖试点城市。坚持经济与环保“双统筹、双促进”，推动大气污染防治深化、细化、常态化，狠抓燃煤、扬尘、机动车、工业、“散乱污”等污染治理。10家燃煤电厂全部完成超低排放治理，累计拆改燃煤小锅炉1300余台，燃煤总量消减980万吨，治理“散乱污”企业10274家，淘汰黄标车和老旧车辆16.5万辆。2017年PM10、PM2.5浓度分别降至118、66微克/立方米，实现年度“两降一增一提升”。今年上半年，空气质量改善幅度位居全国169个地级及以上城市第16位，群众的“蓝天白云”获得感越来越强。“碧水工程”全面开展。按照“安全河、生态河、景观河、文脉河、幸福河”的建设理念和海绵城市标准，加快实施贾鲁河综合治理工程，河道主体工程基本完工，宽阔水面和两岸绿化景观初步形成；索须河生态景观提升工程有序实施，牛口峪引黄工程全线开工，环城生态水系循环、石佛沉砂池至郑州西区生态供水项目一期工程基本完工。河长制、湖长制全面推行，河湖共治共管“一盘棋”治水格局逐步形成。扎实开展“水清河美”专项治理，全国水生态文明城市建设试点任务全面完成，城市水生态环境明显改善。土壤污染防治取得积极成效。围绕重点区域、行业和污染物，实施分类管理和建设用地准入管理，加强农村、农业面源污染防治，推进重点地区重金属总量减排，土壤污染防治和修复不断加强。生态建设步伐加快。以创建国家生态园林城市为抓手，累计新造林34.2万亩，森林抚育改造16.8万亩，新建、完善提升各类森林公园40个，建成“两环三十一放射”等生态廊道3950公里、市级综合公园2个、区级综合公园27个，龙子湖湿地公园和高铁公园建成投用，10个万亩以上森林公园、20个郊野公园、26处生态遗址公园加快推进，建成区绿地率达到35.5%，通过国家园林城市复查。

（八）惠民生增福祉，社会大局和谐稳定。坚持发展为民，以办好省市重点民生实事为抓手，着力弥补民生短板。公共财政民生支出年均增长21.2%，达到1199.5亿元，占公共财政预算支出的79.2%。脱贫攻坚取得决定性进展。把抓好脱贫攻坚作为重大政治任务和第一民生工程，聚焦做好“转、扶、搬、保、救”五篇文章，累计投入财政扶贫资金26.4亿元，全市建档立卡劳动力就业3.04万人，实现就业脱贫2.95万人，易地扶贫搬迁6.1万人，全市贫困发生率由2.12%下降到0.12%，黄河滩区居民迁建进展顺利。就业和社会保障工作持续加强。持续扩大城乡就业，累计接收高校毕业生36.5万人，初次就业率90%以上。全市基本养老保险、基本医疗保险参保人数分别达到648.1万人、759.1万人，城乡居民医保制度整合完成。全市各项社会保险参保1935.4万人项（含退休人员），今年扩面任务提前完成。教育卫生体育事业繁荣发展。大力实施优质教育资源、医疗资源倍增工程，市区新建、改扩建中小学校143所，新建、改扩建幼儿园183所。今年市区新建中小学校18所、幼儿园8所。强化优质教育资源供给，清华附中等一批合作项目落地建设。健康郑州加快推进，公立医院全部取消药品加成，公立医院综合改革受到国务院表彰；分级诊疗制度逐步完善，医疗联合体实现区域全覆盖；区域医疗中心建设持续推进，器官移植和儿童医疗纳入省政府6大国家区域医疗中心建设；全面“两孩”政策平稳实施。民生保障不断加强。城乡低保标准稳步提高，特困供养、孤儿等救助标准增幅22%以上。新建、改扩建养老服务设施460家，新增养老床位1.48万张。“双拥”优抚政策全面落实。全面推行“三房合一”保障制度，新开工保障性住房56.4万套（户），基本建成22.9万套，公租房实物分配入住3.05万户。青年人才公寓建设进展顺利。文化体育事业繁荣发展。颁布实施《郑州市文明行为促进条例》，大力促进公共文明素养提升行动落实。文化事业和文化产业蓬勃发展，文化惠民工程、精品工程深入实施。文艺创作持续繁荣，我市跻身全国最爱阅读城市行列。成功举办世界摩托艇锦标赛、国际摄影大赛等活动。奥林匹克体育中心、文博艺术中心、市民活动中心、现代传媒中心加快建设，城乡公共文化服务体系更加完善，基层公共文化服务设施覆盖率达到95%以上。传承历史文脉、彰显文化特色的商都历史文化区、古荥大运河文化区、百年德化历史文化街区、二砂文化创意园等“四大文化片区”加快建设。成功创建国家首批文化消费试点城市。“扫黄打非”工作进入全国先进城市行列。国家卫生城市通过复审。全民健身与全民健康深度融合，体育事业不断发展。同时，安全生产、食品药品安全、信访稳定、社会治理等工作进一步加强，人民群众的安全感和满意度持续上升。

四年多来，我们自觉执行人大决议决定，主动接受人大监督、政协民主监督和社会监督，累计办理人大代表议案23件、建议2433件，办结率100%、满意率99%；政协提案3115件，办结率100%、满意率99%。加强民主法治建设，推进依法行政，强化行政监察和审计监督，政府自身建设和治理能力不断提升。加强国防教育和国防后备力量及人民防空建设。民族宗教工作创新推进。妇女儿童、残疾人、红十字、慈善等事业健康发展。外事、侨务、对台、统计、史志、气象、地震、社科研究、援疆等工作取得新成绩。

各位代表！四年多来的变革是历史性的、全方位的，变化有目共睹，奋斗凝聚艰辛。这些成绩的取得，是在省委、省政府和市委的坚强领导下，全市人民深入贯彻以习近平同志为核心的党中央决策部署，团结一心、开拓奋进的结果。特别是习近平新时代中国特色社会主义思想，为我们战胜风险挑战、推动经济社会发展指明了前进方向，是我们取得各项成绩的根本所在。在此，我代表市人民政府，向广大工人、农民、知识分子、公安民警及社会各界人士，向驻郑解放军指战员、武警官兵，向关心支持郑州发展的港澳台同胞、海外侨胞和国际友人，致以崇高的敬意和诚挚的感谢！

回顾过去，我们也清醒认识到，经济社会发展中不平衡、不充分的问题依然突出，主要表现在：产业结构调整正处于新旧动力转换期，经济下行压力加大，工业中传统资源型产业比重仍然较高，先进制造业和现代服务业的支撑作用还有待提升；城乡发展方式粗放、建设任务艰巨，基础设施建设滞后于城市的快速发展，城乡二元结构、“大都市病”问题亟待解决；创新基础相对薄弱，高层次的科教机构和创新型、开放型人才相对不足；大气污染防治形势仍很严峻，资源环境约束加剧；社会治理和公共服务事业存在短板，与群众生活密切相关的一些热点问题还没有得到很好的解决，维护社会和谐稳定的任务依然繁重；政府职能转变还不到位，一些领域不正之风和腐败问题仍然不容忽视，等等。对此，我们一定勇于担当，切实解决，决不辜负人民重托！

二、今后五年的目标任务

今后五年，是全面建成小康社会的决胜期，是实现“两个一百年”奋斗目标的历史交汇期。中央明确提出支持郑州建设国家中心城市，把郑州定位为“一带一路”重要节点城市，要求郑州建成连通境内外、辐射东中西的物流通道枢纽，朝着“买全球、卖全球”目标迈进。特别是随着“三区一群”等重大国家战略的落地实施，郑州为内陆地区扩大开放探索路子、为中部崛起提供支撑的责任更加重大。省委、省政府全力支持郑州建设国家中心城市、推动郑州大都市区建设，要求郑州在高质量发展上主动作为，在全省高质量发展上发挥好龙头带动作用，郑州的使命更加光荣。全面建成小康社会，推进郑州更好更快发展，发挥好省会城市的率先带动作用，为中原更加出彩做出更大贡献，让人民群众享受到更多的发展成果，是时代赋予郑州的历史使命。

今后五年的总体要求是：高举习近平新时代中国特色社会主义思想伟大旗帜，全面贯彻落实党的十九大精神，认真落实习近平总书记调研指导河南和郑州工作时的重要讲话精神，积极践行新发展理念，坚持稳中求进工作总基调、奋发有为总要求，全面落实中央、省委、市委各项决策部署，统筹推进“五位一体”总体布局和协调推进“四个全面”战略布局，着力发挥优势打好“四张牌”，突出高质量发展，以国家中心城市建设为统揽，以“三区一群”建设为引领，以现代化国际化生态化为方向，坚持“四重点一稳定一保证”工作总格局，坚持目标导向、问题导向和“创优势、增实力、补短板、能抓住”工作方针，加快建设具有发展活力、人文魅力、生态智慧、开放包容的国家中心城市，努力在决胜全面建成小康社会、全面建设社会主义现代化进程中走在前列，为实现中原更加出彩做出省会城市应有的贡献。

根据市十一次党代会和市委十一届四次、五次、六次、七次全会提出的奋斗目标，建议今后五年经济社会发展的主要目标是：国家中心城市建设取得显著成效，综合实力持续增强，国际枢纽地位基本确立，现代化国际化大都市的框架体系基本形成，高质量、高水平小康社会全面建成，并乘势而上全面推进新时代社会主义现代化建设，对中原更加出彩的辐射带动和全国大局的服务支撑作用更加彰显，人民群众的获得感、幸福感、安全感不断增强。

——综合实力更加雄厚。经济持续健康发展，“三区一群”战略深入实施，产业高端化、绿色化、智能化、融合化水平显著提高，科技实力和创新能力大幅跃升，在确保质量和效益的基础上，生产总值年均增速高于全省平均水平0.5个百分点左右，常住人口城镇化率达到77%以上，中心城市现代化国际化生态化、县域城镇化、城乡一体化迈出更大步伐，乡村振兴取得重要进展，社会文明程度、文化软实力显著提高。

——经济体系更加优化。以先进制造业为支撑、以现代服务业为主导的产业体系基本形成，科技创新体系全面构建，高效市场体系更加完善，良好生态体系初步建立，供给结构与需求结构平衡性、适应性、灵活性显著增强。

——环境质量更加向好。大气污染治理取得决定性成效，PM10、PM2.5平均浓度达到省控目标；水环境质量根本改善，土壤环境质量逐步好转，单位生产总值能耗下降4.4%；新增供热面积2500万平方米，绿化覆盖率达到42%、森林覆盖率达到35%左右，人均公园绿地面积达到12.5平方米，郑州的天更蓝、地更绿、水更清，环境更宜居，生态更美好。

——社会事业更加发展。就业、教育、卫生、文化、体育等基础设施和公共服务体系更加健全，多层次社会保障体系更加完善，基本公共服务均等化水平明显提高，新型住房保障体系、多层次养老服务体系基本形成，民生保障网进一步织密扎牢。

——人民生活更加富裕。全市现行标准下农村贫困人口全部脱贫，城乡居民收入与地区生产总值同步增长，居民生活条件和生活质量全面改善，人民群众幸福感、获得感、安全感更加充实、更有保障、更可持续。

——社会治理更加高效。党的领导核心作用进一步增强，风清气正的政治生态基本形成。法治政府取得明显成效，社会治理社会化、法治化、智能化、专业化水平明显提高，公平正义进一步彰显，安全发展环境更加巩固。

工作中，要始终坚持以习近平新时代中国特色社会主义思想为指导，把握好以下原则：一是坚持党对一切工作的领导。牢固树立政治意识、大局意识、核心意识、看齐意识，自觉做到“四个服从”，坚决维护习近平总书记党中央的核心、全党的核心地位，坚决维护党中央权威和集中统一领导，不折不扣贯彻落实中央、省委和市委决策部署。二是坚持以人民为中心的发展思想。始终站稳群众立场，坚持群众路线，所有工作都要围绕让人们生活更方便、更舒心、更美好来谋划实施，让决策部署更加符合郑州实际、更加符合群众愿望，以务实为民的实际行动凝聚人心、汇聚民力。三是坚持高质量发展。全面贯彻新发展理念，坚持把发展作为第一要务，突出质量第一、效益优先，聚焦质量变革、效率变革、动力变革，加快构建现代化经济体系，推动郑州制造向郑州创造转变、郑州速度向郑州质量转变、郑州产品向郑州品牌转变。四是坚持深化改革开放创新。强化“三区一群”引领带动作用，通过全面深化改革激发内生动力，通过扩大开放增强外源动力，通过强化创新催生新生动力，着力构筑郑州发展新优势。五是坚持国家中心城市建设实践体系。市委明确的“四重点一稳定一保证”工作总格局，坚持目标、问题“两个导向”和“创优势、增实力、补短板、能抓住”工作方针，“十大主攻方向、九大支撑性工程、六大基础性工作”工作部署，“党委加强领导、政府充分履职、人大政协共同参与的责任明晰、工作有序、运转高效”工作推进机制，是我市从实践中探索出来的行之有效的实践体系，我们必须坚持好、完善好、运用好。

三、2018年四季度重点工作

2018年是贯彻落实党的十九大精神的开局之年，是改革开放40周年，是决胜全面建成小康社会、实施“十三五”规划承上启下的关键一年，也是新一届政府工作的起步之年。前三季度，我们认真贯彻中央、省委、市委各项决策部署，在推动经济社会持续稳定健康发展、社会大局和谐稳定的同时，实现各项工作任务与时序同步推进，为完成全年目标任务打下了坚实基础。四季度，我们将紧紧围绕年初确定的各项目标任务和市委十一届七次全会部署的重点工作，统筹稳增长、促改革、调结构、惠民生、防风险，努力推动经济社会高质量发展、国家中心城市高质量建设。

综合考虑前期发展趋势和下步工作导向，建议2018年主要预期目标为：生产总值增长8.5%左右，规模以上工业增加值增长8%左右，固定资产投资增长9%左右，社会消费品零售总额增长11%左右，进出口保持平稳增长，一般公共预算收入增长9%以上，税收占一般公共预算收入75%以上，居民人均可支配收入增长与经济增长同步，常住人口城镇化率提高1个百分点以上，研究与试验发展经费占生产总值达到2%，空气优良天数200天以上，节能减排、环境保护等指标完成国家、省下达任务，供给侧结构性改革取得实质性进展，发展质量效益持续提高，人民群众获得感不断增强。

为实现上述目标任务，四季度要突出抓好以下重点工作：

（一）全面打好打赢三大攻坚战。打好三大攻坚战是重大政治责任，也是决胜全面建成小康社会的底线任务，我们将集中力量、坚决打好打赢，确保完成年度攻坚任务。

坚决打好防范化解重大风险攻坚战。切实增强风险意识，扎实开展防范和处置非法集资、互联网金融风险等专项行动，配合做好影子银行等风险防范工作。摸清政府债务及隐性债务底数，严格落实债务风险化解措施，稳妥化解累积的债务风险，有效规范举债融资，坚决遏制隐性债务增量，切实防范区域性系统性风险。防范化解企业风险和房地产风险，系统开展“问题企业”“问题楼盘”排查化解，切实加强房地产市场秩序综合整治。

坚决打好脱贫攻坚战。紧紧围绕“一手抓脱贫攻坚扫尾，一手抓脱贫成效巩固提升”目标，继续以“N+2”脱贫计划为主线，坚持专项扶贫、行业扶贫、社会扶贫互为补充的大扶贫格局，持续在攻克深度贫困堡垒、提升精准帮扶实效、补齐短板、凝聚合力上下功夫，确保除政策性托底外的3119人存量贫困人口全部脱贫。全面落实省委部署，扎实做好对口帮扶贫困县工作。

坚决打好环境治理攻坚战。深入推进大气污染防治。紧紧围绕年度目标任务，坚持精准施策、标本兼治，按照蓝天保卫战三年作战计划和年度攻坚战“1+10”方案部署，强力推进污染防治攻坚。认真做好中央环保督察“回头看”及省委、省政府环保督察交办问题的整改工作。扎实推进清洁取暖国家示范城市建设，力争煤炭消费总量再下降245万吨；坚持控“尘”、控“煤”、控“排”、控“车”、控“油”、控“烧”并举，强力推进源头治理。突出抓好重点区域治理、重点时段管控、重污染天气应对，坚决杜绝环保“一刀切”行为，推动经济与环保双统筹、双促进。大力实施水污染防治。持续推进水污染防治三年攻坚战，坚持全域治理、系统治理、源头治理，系统推进水污染防治、水生态保护和水资源管理。

加强城市饮用水源地和南水北调中线工程保护，开展城市重点河流水质提标整治，实施污水处理厂提标工程，建成区内河流达到地表水Ⅲ类水质，国控断面I—III类水质比例总体达到53%以上，全部消除建成区黑臭水体。推进实施土壤污染防治。以改善土壤环境质量为核心，年底前完成全市农用地土壤污染详查工作，开展建设用地污染状况普查。加强农村农业面源污染防治。强化土壤污染管控与修复，确保污染地块安全利用率不低于90%、重点行业的重点重金属排放量实现“零增长”。

（二）高质量推进以人为核心的新型城镇化。把握“以建为主、提升品质、扩大成效”的阶段性任务，坚持“中心城市现代化国际化生态化、县域城镇化、城乡一体化”工作主线，高质量推进新型城镇化建设。

进一步完善规划体系。高质量开展新一版城市总体规划编制工作，着力构建“双城引领、多组团、多节点”的大中小城市、中心镇和特色村镇合理布局的城镇体系。加快推进《沿黄河南岸生态文化带空间规划》《郑州历史文化名城保护规划》等重点规划的编制报批工作。加快“1+4”郑州大都市区规划统筹，规划启动郑开双创走廊等重点产业带和生态带重大工程，全面开工机场至许昌市域铁路，重点推进郑汴、郑许一体化，郑新、郑焦深度融合发展。统筹谋划中心城区与周边组团新区、县（市）的联动融合，推动区域均衡协调发展。启动科学大道科学谷核心区建设。

进一步强化现代化国际性立体综合交通枢纽地位。配合做好郑济、郑万、郑合高铁建设，加快推进郑州南站及配套建设。全力推进新郑机场至郑州南站城际铁路等项目建设，启动占杨、薛店铁路物流基地建设。高质量编制新一轮《“畅通郑州”规划白皮书（2019—2021）》，完善高速公路等骨干路网规划，加快构建以地铁、快速路网为主体的城市立体交通体系。加快“轨道上的郑州”建设，力争轨道交通三期规划尽早获批；年底前，轨道交通2号线二期等线路主体结构完成，5号线实现空载试运行，全面推进6号线市政配套工程建设；力争轨道交通累计客运量突破7.5亿人次。加快推进“井字+双环”城市快速路网、中心城区和外围组团连接通道建设，加快四环线及大河路快速化、农业路铁路代建段等重点工程建设，确保北三环东延高架主线、东三环隧道建成通车，力争金水路西延主线贯通；打通一批支线路网。加快环城货运通道建设，开工建设国道310郑州西南段改建工程、省道312郑州境改建工程，加快推进国道310中牟境改建工程，建成国道107郑州境东移（一期）工程。确保国家“公交都市”示范城市创建成功；加快推进公共交通城乡一体化，研究推行域内高速公路快速高效通行措施。

完善提升城市功能。大力实施水电气暖污等基础设施扩容提质，开工建设桥南水厂、龙湖水厂，侯寨水厂具备通水条件，新增日供水能力10万吨。推进西气东输薛店分输站—航空港区天然气管道工程建设。加快新力电力“热电外迁”，力争年底前建成投运；推进裕中电厂“引热入郑”集中供热管网及配套设施建设，新增供热500万平方米。完成马头岗污水处理厂一期、南三环中水公园等污水污泥处理工程建设。加快垃圾焚烧发电厂建设。

加快保障性安居工程建设。围绕“两个大头落地”目标任务，加快安置房建设及群众回迁安置进度，10月底前已规划安置地块全部实现实质性开工，确保全年新开工棚户区改造安置房2.15万套、保障性安居工程基本建成6万套、回迁群众20万人、网签安置房8万套以上。提升安置房建设品质，同步做好市政设施及公共服务设施建设，确保与安置房同步交付使用。加快青年人才公寓建设，二期2万套全部开工。

深入推进百城建设提质工程。加快老旧片区建设提质和示范区建设，按照“共同缔造”理念，重点探索组织运作、资金筹措、项目建设和长效管理“四个机制”，最终形成可复制、可推广的工作经验，把老旧片区建设提质建成民心工程、群众满意工程。加快推进城市“双修”、综合管廊、城市设计、海绵城市试点城市建设；新建公共建筑全部落实绿色建筑标准。

强力推进城市精细化管理。围绕理念、职能、机制转变和标准提升“三转一提”，深化城市管理体制改革，推动管理重心下移，建立健全“路长制”管理模式，持续提升城市精细化管理水平，打造安全、整洁、有序、文明的城市环境，营造“人民城市人民建、人民城市人民管”共建共享共治的浓厚氛围。对市区192条道路（路段）开展大中修，对71条支路背街实施改造提升，实现机扫作业路段全覆盖。推进生活垃圾分类管理，完成东、西餐厨垃圾厂建设，年底前分类覆盖率达到30%以上、无害化处理率达到100%。新建公共停车泊位不少于5万个。加快推进“厕所革命”，中心城区新建公厕1200座，升级改造765座。加大共享单车、渣土运输车等特殊车辆综合治理。以“洁化、亮化、美化”为目标，强力推进楼顶整治工作。外迁或提升大围合区域商品交易市场31家。

全面加快新型智慧城市建设。坚持“善政、惠民、兴业”的建设方向，按照《推进新型智慧城市建设的实施意见》和《郑州市新型智慧城市建设三年行动计划工作推进方案》，加快实施智慧城管、智慧环保、智慧医疗、智慧停车、智慧社区、智慧政务和“一卡通”“i郑州”等重点示范应用项目，不断满足社会公众对高品质智慧生活的需要。

加快城乡一体化进程。扎实做好乡村振兴战略规划编制工作，持续开展农村人居环境整治行动。开展扶持壮大村集体经济、农村公共服务试点工作，加快城乡融合共享特色田园乡村综合体建设；高标准高水平推进美丽乡村试点村项目30个。全面清理整治以设施农业名义开发建设“大棚房”等违法占地行为，确保我市耕地和永久基本农田保护面积不低于省政府下达的目标。

（三）高质量构建现代产业体系。深入贯彻习近平总书记“三个转变”重要论述，以高端化、智能化、绿色化、融合化为方向，围绕12个转型升级产业、5个重点攻坚产业，集中力量在观念、结构、体制、创新等四个方面实施攻坚。

推动制造业高质量发展。以建设中国制造强市为引领，加快培育世界级产业集群，持续提升电子信息、汽车、装备制造等六个千亿级产业能级，推进制造业高质量发展、向中高端迈进。突出招大引强和项目建设，争取再签约引进一批电子信息、装备制造等主导产业重大项目；加快推进151个市级、376个县区级重大项目建设，力争全年完成工业投资1400亿元。持续推进“三大改造”，力争全年推动规模以上工业企业完成技术改造700家。实施工业研发投入三年倍增计划，完善制造业创新体系。深入实施企业家领航计划、大企业培育和百家高成长性企业三年倍增计划，全年力争新增百亿元企业2家、规模以上企业150家。积极创建全国工业互联网示范城市，加快两化融合管理体系贯标，加快郑州信息安全产业基地建设，推动100家规模以上工业企业和1000家企业上云平台。加快郑州军民融合产业创新基地、河南（巩义）军民融合产业基地建设。实施加快制造业高质量发展“1+N”系列政策，强化开发区、县（市）和上街区工业主导地位，加快主城区服务型制造和生产性服务业发展，推动制造业向高端化、集群化、智能化、融合化、绿色化迈进。注重产业集聚区提质增效，突出抓好产业转型、创新驱动、绿色集约、产城融合“四大发展”任务，确保产业集聚区固定资产投资和规模以上工业主营业务收入分别增长10%。

促进服务业提质增效。加快服务业“两区”建设，壮大产业规模、形成集群优势，提升业态档次。以消费升级为导向，力争全年服务业增加值增长9.5%。进一步完善金融服务体系，力争全年金融业增加值突破1100亿元。加快中原基金岛建设，基金管理规模力争达到3000亿元。加快国家大数据综合试验区核心区建设，力争中科院计算技术研究所、中国信通院、紫光云数等项目年内落地。加快建设国家现代物流创新发展试点城市，做大做强冷链、快递、电商等特色物流，壮大物流业总部经济，深入开展供应链体系建设，为现代物流业成为我市新经济增长点和产业支撑打下坚实基础。持续推进引导城乡居民扩大文化消费试点工作，推动旅游业转型升级，实施“沿黄旅游带”、海昌极地海洋公园、宋城·黄帝千古情等重大项目。以我市获批全国住房租赁试点城市为契机，进一步加大房地产市场调控力度，加快构建多主体供给、多渠道保障、租购并举的住房制度，推动房地产业持续稳定健康发展。抓好《加快建设郑州健康养老产业实施方案（2018—2020）》贯彻落实，推动健康养老产业繁荣发展。

做优都市生态农业。加快都市生态农业和绿色食品业转型升级，大力实施绿色标准化可追溯“菜篮子”、绿色食品加工业提升、生态农业建设和休闲农业星级农庄创建四大工程，着力提升速冻食品、面制品、肉制品等六大优势产业，力争全年农产品加工总产值达到2150亿元，全市休闲农业营业收入达到38亿元，接待人次超过3400万。

支持非公有制经济加快发展。扎实贯彻省促进非公有制经济健康发展大会精神，大力支持民营企业发展，出台支持非公有制经济发展的政策措施，让非公有制企业享受到更多政策红利；积极构建亲清新型政商关系，努力实现非公有制经济创新发展、转型发展、提质发展、高质量发展。

（四）高质量实施开放创新双驱动。坚持把创新作为引领发展第一动力，把巩固提升开放优势作为战略突破口，加快构筑高质量发展的战略支撑，持续为经济社会发展注入更多动力活力。

加快构建全方位对外开放新格局。抢抓新一轮对外开放战略机遇，高质量建设“三条丝路”、无缝衔接“海上丝绸之路”，高标准打造开放型经济新体系，高水平建设河南自贸区郑州片区，高密度集聚开放创新资源，高品质提升城市国际化水平，着力把郑州建设成为国家内陆地区对外开放门户。强力推进开放平台建设。加快机场三期建设，扎实推进郑州—卢森堡“空中丝绸之路”建设，开通郑州—卢森堡洲际直飞客运航线，确保新郑机场全年客货运吞吐量分别突破2700万人次、55万吨。加快在航空港实验区建设全国重要的飞机租赁业集聚地；积极推进郑州国家通航产业综合示范区建设，高标准构建以上街机场为平台的通航机场群网。加快《中国（河南）自由贸易试验区郑州片区空间布局规划（2017—2035）》编制工作。扎实落实河南自贸区郑州片区256项改革试点任务，力争形成一批创新经验并复制推广，积极申建自由贸易港。加强各类口岸和海关特殊监管区建设，力争汽车口岸二期主体建成，全面落实推进7×24小时通关制度。加强铁路口岸与沿海港口合作，建设东向、南向等海铁联运国际通道。推进中欧班列（郑州）创新发展，力争全年开行650班以上。加快郑州国际陆港第二节点规划建设，形成新丝绸之路经济带上的“陆港金桥”。深入推进跨境电商综试区建设，高水平建设EWTO核心功能集聚区。以第13次中欧区域政策合作研讨会、“一带一路”沿线国家海关贸易便利化高级研讨班为契机，进一步深化对外合作。持续加大招商引资力度，聚焦产业发展关键领域，着力在集聚区建设、价值链提升、区域协同发展等方面取得突破，努力构建“四位一体”招商网络，确保73个“五职”招商项目当年签约、当年开工。

大力推进创新型城市建设。加快建设国家自主创新示范区，积极推进“一谷一中心、五区二十园”建设，加大推进核心区人事、薪酬等体制机制改革力度，及时总结改革经验，适时向全市推广。探索创新区域产业发展合作共赢模式，新引进培育国内外知名技术转移机构2家，新建科技公共服务平台5家，确保全市研发经费支出大幅提高，力争专利申请量、授权量分别达到5.7万件、2.6万件。力争全年培育高新技术企业300家，引进领军人才和高层次紧缺人才200名，培育企业建设省级各类创新平台100家。持续推动大众创业万众创新，高质量建设国家级和省级“双创”示范基地，继续实施众创空间倍增、创客育引等六大工程，全年新建各类创新创业载体20个，新增入孵企业（团队）1000家。

（五）持续深化重点领域改革。以改革开放40周年为契机，积极主动推进重点领域改革，推动重点难点任务取得新突破。全面落实政府机构改革相关任务。贯彻中央、省委、市委机构改革决策部署，优化政府机构设置和职能配置，严格按照时间节点，全面完成各项工作任务。深入推进供给侧结构性改革。坚持运用市场化法治化手段，化解钢铁、煤炭、水泥、电解铝等重点行业过剩产能，完成省下达的煤炭行业化解过剩产能任务；淘汰全部不符合要求的落后产能。妥善分流安置职工和处置资产债务，妥善处置特困企业。大力支持实体经济发展，落实好优惠政策，建立降成本长效机制，着力降低企业融资、制度性交易、人工、要素、物流等成本。加快建设国际化营商环境。围绕世界银行10项重要指标，大力实施“企业开办注销”提速等十大专项提升行动，力争用2~3年时间，使我市营商环境的国际化、法治化、便利化水平大幅提升，各领域营商环境便利度全面进入国内先进行列，跨境贸易、开办企业等部分指标达到国际先进水平，将郑州建设成为国际接轨、国内领先、中部一流的营商环境新高地。深化“放管服”改革。扎实做好“三级十同”梳理规范，对照省级通用目录和事项清单，按照“N±X”要求，10月底前梳理完成市县乡目录和清单。持续推进“一网通办”，按照“真联、真通、真办”要求，全面对接省政务服务网，确保10月底前所有非涉密依申请行政权力事项80%以上实现网上申报，年底前所有非涉密事项开通网上申报。力争年底前投资项目审批时间压缩至100个工作日以内，不动产交易与登记全流程5个工作日以内，确保超前完成省定改革目标。提升实体大厅建管水平，按照“三集中、三到位”要求和“应进必进”原则，确保部门、事项、人员顺利实现集中进驻，全面实施“容缺受理”“一口受理、集成服务”，为群众办事提供“只进一扇门”“最多跑一次”的“一站式”服务。深入推进国企改革。紧紧扭住“僵尸企业”处置“牛鼻子”、持续加大剥离企业办社会职能力度、全面加快市直部门监管的市属企业改革、紧盯市管工业企业改制退出目标任务，持续深化企业产权制度改革、企业组织结构改革和法人治理结构改革。同时，积极做好财税体制、投融资体制和农村综合改革；加快社会信用体系建设。

（六）高质量推进生态文明建设。深入贯彻习近平生态文明思想，以更大力度、更实措施推进生态文明建设，加快形成绿色生产方式和生活方式，切实让群众不断感受到生态环境的改善。优化生态功能布局。统筹考虑人口资源环境承载能力，科学划定“三区三线”，着力打造“三圈、一带、九水、三十一廊”的网络化生态空间格局。持续加快生态绿化建设。推进实施沿黄生态防护林带景观提升、湿地生态系统修复保护、生态廊道网络提升等工程；加快郑州黄河国家湿地公园、郑州野生动物园、郑州新植物园、西流湖公园等公园游园建设；加快郑东新区象山等九大山体公园建设；提升提质全市公园和树木园，不断提高300米见绿、500米见园覆盖率。扎实推进生态保遗工程。切实抓好水生态文明建设。全面深化落实河长制、湖长制，以一河一湖一策为抓手，实现管水治水常态化长效化。年底前，贾鲁河综合治理蓝线工程基本建成，绿线工程绿化、铺装等工程全面启动；牛口峪引黄、环城生态水系循环和石佛沉砂池至郑州西区生态供水等水源工程建成通水；力争郑州市西水东引工程开工建设；积极谋划郑汴洛水系连通等大水源工程，着力构建“循环通畅、丰枯调剂、蓄泄兼筹、多源互补”的现代水系。完善生态文明制度体系。继续推动国家生态文明先行示范区建设，探索开展全市生态文明建设目标评价考核工作，健全源头预防、过程控制、损害赔偿、责任追究的生态文明制度体系。

（七）高质量保障和改善民生。着力解决结构性民生问题、实施普惠性民生工程、办好紧迫性民生实事，努力在幼有所育、学有所教、劳有所得、病有所医、老有所养、住有所居、弱有所扶上取得新进展。扎实办好重点民生实事。强化责任意识和为民意识，对承办的省市重点民生实事进展情况开展“回头看”，确保到年底各项任务不折不扣完成。千方百计扩大就业。突出做好高校毕业生、城镇困难人员、去产能职工等重点群体就业工作，实施全民技能振兴工程，确保全年实现新增城镇就业11万人，完成农村劳动力转移就业5万人。完善社会保障体系。不断健全社会救助体系，支持老龄事业、社会福利和慈善事业发展，兜牢民生底线。实施全民参保登记计划，进一步提高养老保险待遇水平。推行多元复合式医保支付方式，深入推进异地就医联网结算。加快教育卫生事业发展。继续实施第三期学前教育行动计划，着力解决入园难、入园贵、监管弱等问题；市区新建改扩建中小学校30所以上，开工建设普通高中学校4所，逐步消除“大班额”，推动城乡义务教育一体化发展。深入实施职业教育攻坚二期工程。加大市属高校建设支持力度，确保郑州师范学院通过教育部合格评估；加快引进优质高等教育资源，支持郑州大学“双一流”建设。统筹推进“三医联动”改革，加快分级诊疗制度和医疗联合体建设，全面推动家庭医生智能化签约服务；加快儿童、器官移植等区域医疗中心建设，支持中医药传承发展，优化健康服务供给模式。繁荣发展文化体育事业。巩固全国文明城市创建成果，全力推进商都历史文化区等“四大文化片区”建设，确保年内全部具备实质性开工条件；高标准加快建设郑州中央文化区（CCD)，要抢抓工期，全面加快“四个中心”建设，确保到年底，奥体中心、市民活动中心和美术档案史志馆、大剧院项目完工，博物馆、报业大厦完成外立面装修，广播中心主体结构封顶、电视中心完成地下工程；市民文化服务区南区等20项续建项目完成，同时尽快启动北部片区建设。完善公共文化服务体系，确保80%以上的基层综合性文化服务中心年底前完成建设任务。统筹推进全市历史文化名城保护和申报工作。加强城乡公共体育设施建设管理，打造城市社区“15分钟健身圈”；做好第十一届全国少数民族运动会筹备工作，积极申办国际A级体育赛事。持续加强社会治理。深入开展“扫黑除恶”专项斗争，持续做好反恐体系建设、社会治安、信访稳定、安全生产、食药安全等工作，加强互联网管理，强化矛盾排查化解，确保社会大局和谐稳定。

加强国防动员、国防后备力量和人民防空建设，推动军民融合深度发展。支持驻郑解放军、武警部队建设，深化“双拥”共建工作。持续推进民族团结进步创建活动，促进宗教和谐和睦。全面做好统计、审计、地方志、外事侨务、对台事务、社科研究、档案管理、防震减灾、援疆等工作。

进一步提高政治站位，明确责任，细化方案，严明措施，切实把中央、省委巡视反馈的“统筹协调力度不够、国家中心城市建设未形成强劲势头”等9个方面18项具体问题、国务院大督查中发现问题、中央扫黑除恶督导组反馈问题整改到位。同时，按照市委决策部署，及早谋划明年工作。

四、全面加强政府自身建设

迈向新时代、踏上新征程，需要我们担当新使命，展现新作为。未来五年，新一届政府将勤勉履职，锐意进取，严格落实全面从严治党要求，不断加强自身建设，深入推进职能转变，加快建设顺应人民群众期待、适应国家中心城市建设要求的新时代人民满意政府。

（一）加强政治建设，增强“四个意识”。自觉以习近平新时代中国特色社会主义思想为指引，增强“四个意识”、做到“四个服从”，坚决维护习近平总书记党中央的核心、全党的核心地位，坚决维护党中央权威和集中统一领导，始终同以习近平同志为核心的党中央保持高度一致，推动中央方针政策、省委省政府工作部署在郑州落地生根。推动“两学一做”学习教育常态化制度化，坚定理想信念，自觉在推动发展、服务人民中提升工作水平。

（二）坚持依宪施政，推进依法行政。严格遵守宪法法律，加快建设法治政府，把政府工作全面纳入法治轨道。依法履行职责，深入实施“七五”普法规划，不断提高运用法治思维和法治方式推动工作的能力。坚持严格规范公正文明执法，有权不可任性，用权必受监督。认真执行市人大及其常委会的决议决定，自觉接受人大法律监督、工作监督和政协民主监督。全面推进政务公开，加强舆情应对，主动回应社会关切。坚持科学、民主、依法决策，对涉及重大公共利益、群众切身利益的事项，主动听取各方面意见，真正使政府工作体现人民意愿、结果由人民评判。

（三）恪守公仆本色，坚持廉洁底线。不忘初心、牢记使命，始终把人民对美好生活的向往作为根本追求。严格落实全面从严治党主体责任和“一岗双责”，健全行政权力监督制约机制，从严查处侵害群众利益行为，保持惩治腐败“零容忍”高压态势。严格贯彻落实中央八项规定及其实施细则精神，坚决防止“四风”反弹回潮，纠正形式主义、官僚主义顽症。加强巡视巡察和审计结果运用，畅通社会监督、媒体监督渠道，加大对权力运行的规范制约。严格执行廉洁准则和纪律处分条例，强化监督执纪问责，着力打造干部清正、政府清廉、政治清明的良好生态。

（四）加快作风转变，强化责任担当。聚焦解决动力不足“不想为”、能力不足“不会为”、担当不足“不敢为”等突出问题，切实增强政府工作人员的政治担当、历史担当、责任担当，大力教育引导公务员在其位、谋其政、干其事、求其效。围绕建设高素质专业化干部队伍，建立激励机制和容错纠错机制，激发广大公务员见贤思齐、积极作为。大力加强干部队伍思想教育，强化专业知识、专业能力培训，注重培养专业作风、专业精神，涵养担当作为的底气和勇气，着力增强适应新时代发展要求的能力，努力以新担当新作为创造新业绩。

各位代表！

“幸福都是奋斗出来的”，实现伟大目标需要坚忍不拔的努力。让我们更加紧密地团结在以习近平同志为核心的党中央周围，在省委、省政府和市委的坚强领导下，乘着新时代的浩荡东风，加满油、把稳舵、鼓足劲，以时不我待、只争朝夕的精神投入工作，全心全意为人民谋幸福，尽职尽责为郑州谋发展，在实现中原更加出彩的征程中，奋力谱写郑州高质量建设国家中心城市的新篇章！
